# TGF-**β**–mediated epithelial-mesenchymal transition of keratinocytes promotes fibrosis in secondary lymphedema

**DOI:** 10.1172/jci.insight.192890

**Published:** 2025-07-29

**Authors:** Hyeung Ju Park, Jinyeon Shin, Ananta Sarker, Mark G. Klang, Elyn Riedel, Michelle Coriddi, Joseph H. Dayan, Sarit Pal, Babak J. Mehrara, Raghu P. Kataru

**Affiliations:** 1 Plastic and Reconstructive Surgery Service, Department of Surgery,; 2 Research Pharmacy, and; 3 Biostatistics Service, Department of Epidemiology and Biostatistics, Memorial Sloan Kettering Cancer Center, New York, New York, USA.

**Keywords:** Cell biology, Dermatology, Inflammation, Fibrosis, Lymph, Skin

## Abstract

Secondary lymphedema is characterized by fibrosis and impaired lymphatic function. Although TGF-β is a key regulator of fibrosis in this disease, the cellular mechanisms regulating this process remain unknown. Epithelial-mesenchymal transition (EMT), a mechanism by which TGF-β induces fibrosis in other skin diseases, is characterized by loss of epithelial cell markers and cellular polarity, upregulation of fibrotic gene expression, and gain of migratory capacity. Using clinical lymphedema biopsy specimens and animal models, we show that keratinocytes in the basal layer of the epidermis undergo EMT in lymphedematous skin, migrate into the dermis, and contribute to dermal fibrosis. In vitro studies using cultured primary human keratinocytes treated with lymphatic fluid from the affected limbs of patients with secondary lymphedema resulted in a TGF-β–mediated increased expression of EMT markers. We show for the first time that EMT is activated by TGF-β in secondary lymphedema and that this process plays an important role in regulating skin fibrosis in this disease.

## Abstract 2

Secondary lymphedema is characterized by fibrosis with unknown mechanisms. Here, for the first time, we show that keratinocytes under go epithelial to mesenchymal transition (EMT) contriobuting to skin fibrosis in lymphedema in a TGF-b dependent fashion.

## Graphical abstract

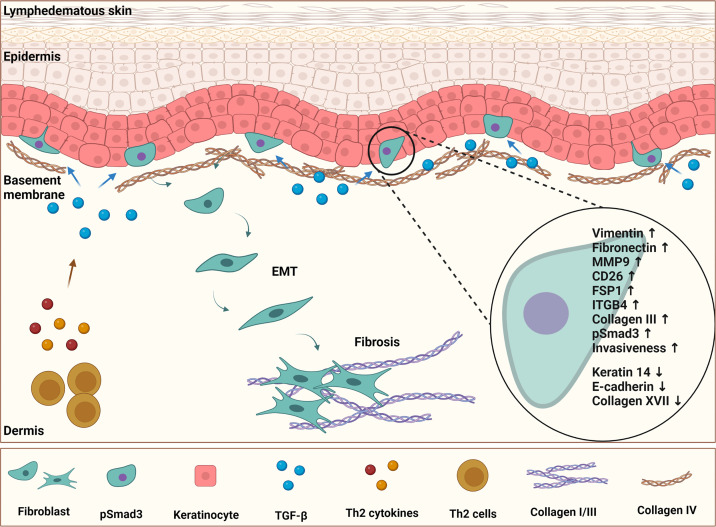

## Introduction

Fibrosis, the excessive production of fibrous connective tissue, is a response to dysregulated wound healing or chronic inflammation. Although collagen deposition is initially beneficial for minor tissue repair, severe or repetitive tissue injury leads to persistent extracellular matrix (ECM) accumulation resulting in organ failure in chronic kidney disease, idiopathic pulmonary fibrosis, cirrhosis, heart failure, and scleroderma (1–10). While recent studies suggest that fibrosis plays a key role in the pathophysiology of secondary lymphedema, the cellular mechanisms regulating this response remain unclear.

Tissue biopsies from patients with secondary lymphedema demonstrate fibroblast proliferation, fibroadipose deposition, and accumulation of ECM products (11, 12). This response is driven by increased expression of transforming growth factor β (TGF-β) and T helper type 2 (Th2) cytokines, including IL-4 and IL-13 (11, 12). Notably, TGF-β and Th2 cytokines are also central to other fibrotic skin disorders, such as scleroderma and atopic dermatitis, as well as in pathological fibrosis of other organ systems (1, 13, 14). Animal models of secondary lymphedema further suggest an interplay between Th2 cytokines and TGF-β signaling pathways, underscoring their importance in lymphedema-induced fibrosis (15, 16). This is supported by the finding that inhibition of TGF-β or Th2 cytokines reduces ECM accumulation, tissue stiffness, fibroblast proliferation, and collagen deposition (11, 17).

Epithelial-mesenchymal transition (EMT), a key regulator of fibrosis in the skin and other organ systems, transforms epithelial cells into ECM-producing mesenchymal cells (18–20). During EMT, epithelial cells lose polarity, downregulate expression of epithelial genes (e.g., cytokeratin and junctional proteins), and upregulate expression of mesenchymal markers (e.g., vimentin, FSP1, fibronectin, collagens, and α-smooth muscle actin) (21). EMT also enables epithelial cells to degrade basement membrane molecules and migrate into underlying tissues (20). TGF-β–induced EMT is a major driving force of fibrosis in fibrotic disorders (22). Furthermore, TGF-b and Th2 cytokines promote fibroblast proliferation, myofibroblast differentiation, ECM production, and the expression of fibroblast-associated genes (e.g., vimentin and fibroblast-specific protein 1 [FSP1]) (2, 3, 23–26). Although TGF-β is known to increase fibroblast proliferation and ECM production in lymphedematous skin, its role in regulating EMT and the resulting contributions to lymphedema-associated fibrosis are unknown.

In this study, we used clinical secondary lymphedema biopsy samples and animal models to test the hypothesis that TGF-β induces EMT of keratinocytes. We provide the evidence that keratinocytes in the basal layer of the epidermis undergo EMT in lymphedematous skin, migrate into the dermis, and contribute to dermal fibrosis. In vitro studies demonstrate that exposure of primary human keratinocytes to lymphedema fluid collected from the lymphedematous limb of patients with secondary lymphedema increases the expression of EMT genes in keratinocytes, and that this process is dependent on TGF-β signaling. Inhibition of TGF-β or Th2 cytokines in preclinical mouse models and in tissues collected from patients treated with neutralizing antibodies against IL-4/IL-13 reduces keratinocyte EMT.

## Results

### Lymphedema results in skin fibrosis and keratinocyte EMT.

**Figure 1:**
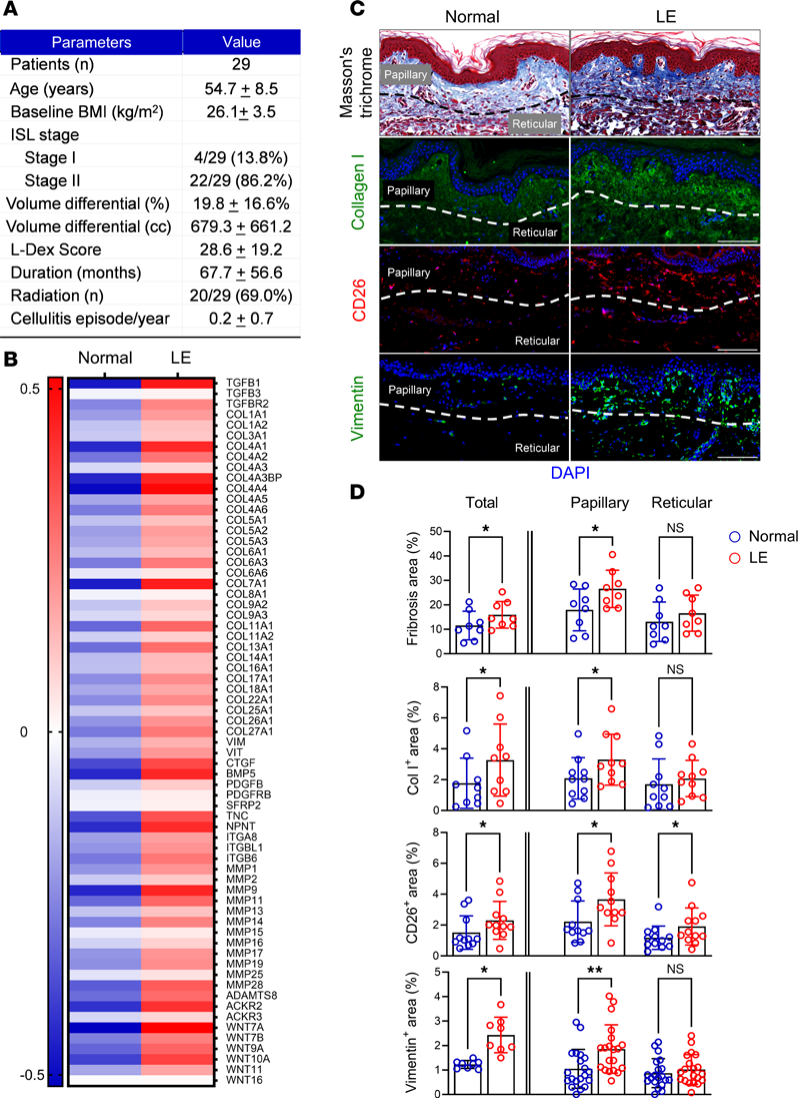
Lymphedema induces skin fibrosis in patients with unilateral BCRL. (**A**) Patient demographics. Data are presented as mean ± SD unless noted. BMI, body mass index; ISL, International Society of Lymphology. (**B**) Expression of fibrosis-related genes by RNA-seq in normal and lymphedematous (LE) skin biopsies (*N* = 4). Each box represents the average mRNA expression of 4 patients. (**C**) Representative image of Masson’s trichrome staining and immunofluorescent staining of collagen I, CD26, and vimentin in normal and LE skin biopsies. Dashed lines delineate papillary and reticular dermis. Scale bar: 100 μm. (**D**) Quantification of fibrosis, collagen I^+^, CD26^+^, and vimentin^+^ areas in normal and LE skin biopsies. Each circle represents the average quantification of 3 high-power field views for each patient (*n* = 8–23). *P < 0.0, **P < 0.01. *P* values were calculated by paired Student’s *t* test. Error bars represent mean ± SD.

To investigate fibrotic skin changes in clinical lymphedema, we compared matched biopsies obtained from the normal and lymphedematous arms of 29 patients with stage I–II breast cancer–related lymphedema (BCRL) (Figure 1A). Bulk RNA sequencing (RNA-seq) analysis comparing the affected arms of 4 patients with their normal limb revealed increased expression of fibrotic genes including vimentin, TGF-βs, platelet-derived growth factor β, collagens, and matrix metalloproteinases (MMPs) in lymphedematous skin (Figure 1B and Supplemental Table 1; supplemental material available online with this article; https://doi.org/10.1172/jci.insight.192890DS1). Histology confirmed increased fibrosis showing collagen accumulation and a higher number of CD26^+^ or vimentin^+^ fibroblasts in the papillary dermis (Figure 1, C and D).

**Figure 2:**
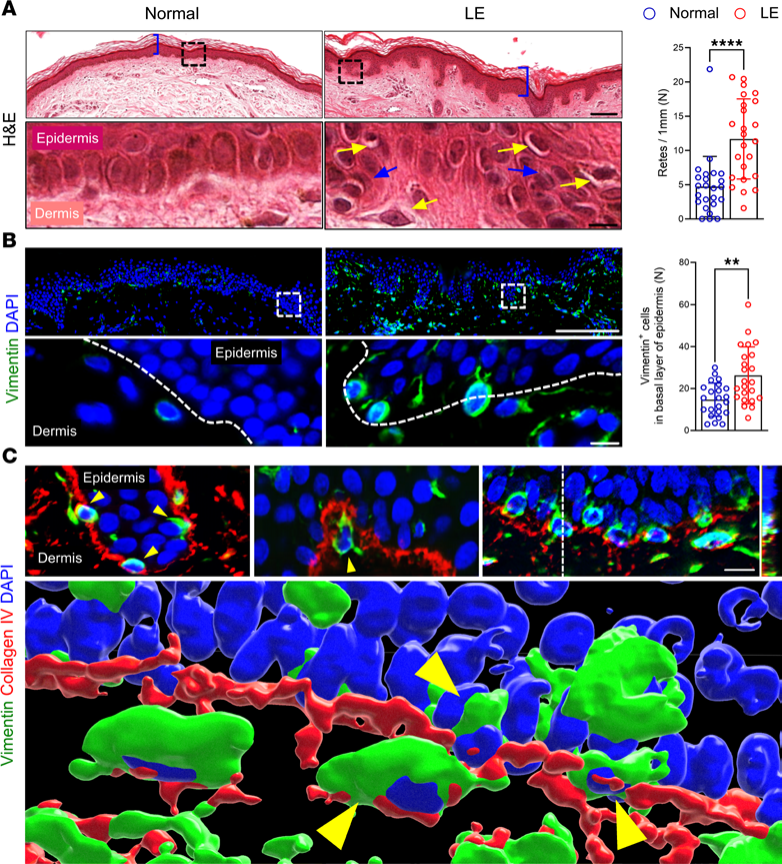
Lymphedema disrupts epidermal architecture and induces vimentin expression in keratinocytes. (**A**) Representative H&E images (scale bar: 100 μm) and magnified views (scale bar: 10 μm) of normal and lymphedematous (LE) skin. Dashed boxes indicate magnified areas. Blue parentheses indicate hyperkeratosis, yellow arrows indicate spongiosis, blue arrows indicate cells with loss of columnar morphology. Bar graph quantifies the number of rete ridges per field. Each circle represents 1 patient (*n* = 25). ****P < 0.0001 by paired Student’s *t* test. (**B**) Immunofluorescence images (scale bar: 100 μm) and magnified views (scale bar: 50 μm) of vimentin staining in normal and LE skin. Dashed lines delineate the epidermis and dermis. Bar graph quantifies vimentin^+^ cells per high-power field (*n* = 23). **P < 0.01 by paired Student’s *t* test. Error bars represent mean ± SD. (**C**) Immunofluorescence of vimentin (green) and collagen IV (red) in LE skin (scale bar: 100 μm). 3D rendering highlights vimentin^+^ cells (arrowheads) within or migrating through the basement membrane.

Keratinocytes in the basal layer of the epidermis in lymphedematous skin exhibited hallmarks of EMT, including hyperkeratosis, spongiosis, and increased rete ridges (Figure 2A). These cells also had markedly abnormal nuclei, loss of columnar morphology, and abnormal cellular polarization (Figure 2A). A large proportion of abnormal basal keratinocytes in lymphedematous skin expressed vimentin as compared with control biopsies (Figure 2B). This is important because vimentin is a cellular marker of EMT that also controls the expression of other EMT genes (27). 3D rendering of confocal images showed vimentin^+^ cells in close association with, and sometimes migrating through, the basement membrane, as denoted by type IV collagen staining (Figure 2C).

### Keratinocytes express EMT-related genes.

**Figure 3:**
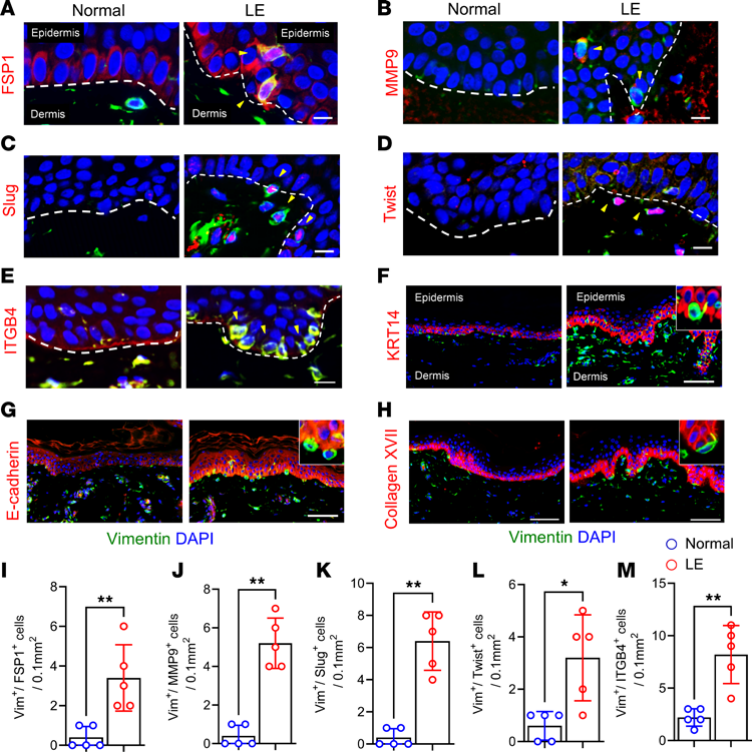
Vimentin^+^ epithelial cells in lymphedematous skin express EMT genes and lose epithelial markers. Representative immunofluorescence images of normal arm skin and lymphedematous arm skin biopsies from patients with unilateral BCRL. Costaining for vimentin (green) and the following markers (red) is shown **A,** FSP1; **B,** MMP9; **C**, Slug; **D,** Twist; **E,** ITGB4; **F**, KRT14; **G,** E-cadherin; and **H**, collagen XVII. Arrowheads highlight vimentin^+^ cells expressing EMT-related genes. Dashed lines delineate the epidermis and dermis. Scale bar: 100 μm. Quantification of EMT markers on Vimentin+ cells **I**, FSP1; **J**, MMP9; **K**, Slug; **L**, Twist; and **M**, ITGB4 from 200 x high magnification images from a 0.1 mm^2^ area. (*n* = 5). *P < 0.05 and **P < 0.01 by paired Student’s *t* test.

Immunofluorescent staining confirmed that significantly high number of vimentin^+^ epidermal cells in lymphedematous skin expressed other EMT markers, including FSP1, MMP9, Slug, Twist, and ITGB4 compared with normal skin (Figure 3, A–E and I–M). Some cells expressed these markers without coexpression of vimentin. Conversely, these cells showed decreased expression of epithelial markers keratin14 (KRT14), E-cadherin, and collagen XVII (Figure 3, F–H).

### Mouse tail lymphedema model recapitulates keratinocyte EMT.

**Figure 4:**
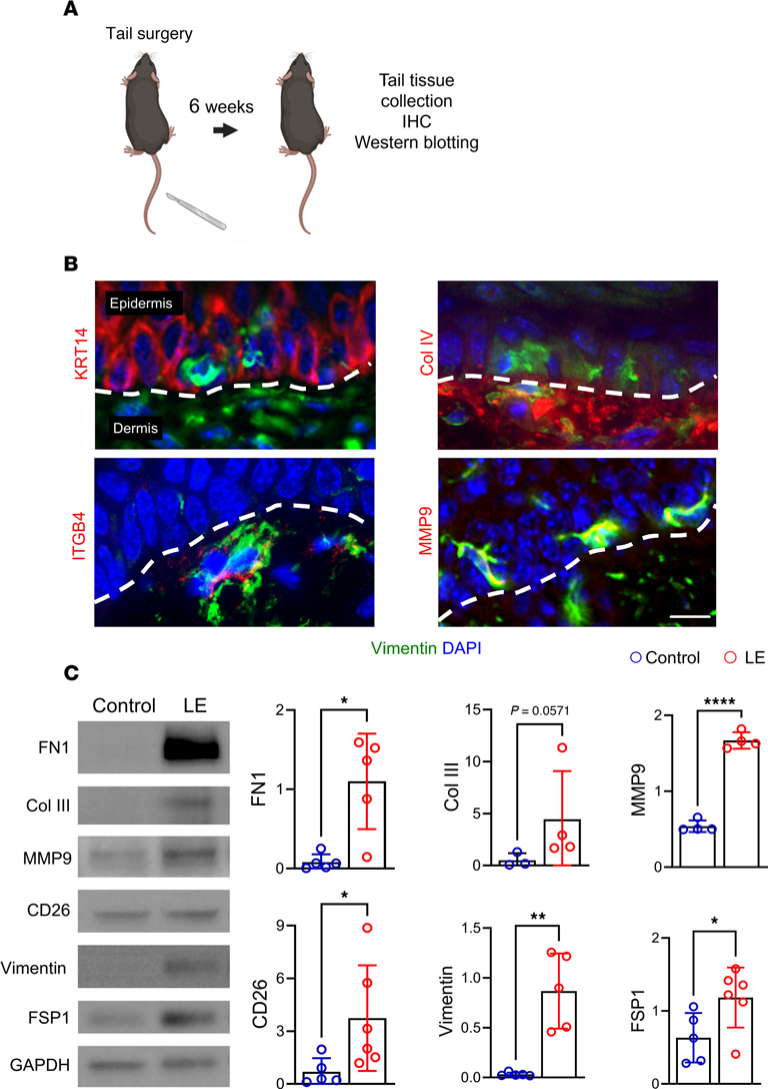
Mouse tail lymphedema induces EMT, mirroring human pathology. (**A**) Schematic illustrates the model and experiment plan. (**B**) Immunofluorescence images (scale bar: 100 μm) show costaining of vimentin (green) and KRT14, collagen IV, ITGB4, or MMP9 (red) in lymphedematous (LE) tail skin. Dashed lines delineate the epidermis and dermis. Note the presence of vimentin^+^ cells within the epidermis and the coexpression of EMT markers. (**C**) Representative Western blot and quantification graphs (relative to GAPDH; *n* = 3–6) demonstrate increased expression of fibronectin, collagen III, MMP9, CD26, vimentin, and FSP1 in LE tail skin compared with control. *P < 0.05, **P < 0.01, ****P < 0.0001 by Mann-Whitney test. Error bars represent mean ± SD.

The mouse tail lymphedema model mirrored the EMT observed in clinical samples. Six weeks after tail skin and lymphatic excision, lymphedematous tail skin showed increased vimentin^+^ epidermal cells, increased expression of EMT-related genes, and decreased expression of epidermal markers (Figure 4, A and B, and Supplemental Figure 1). Western blot analysis of isolated epidermis confirmed the upregulation of fibronectin (18.3-fold), MMP9 (3.1-fold), CD26 (5.4-fold), vimentin (36.2-fold), and FSP1 (1.9-fold) in lymphedematous samples (Figure 4C).

### Lineage tracing confirms EMT and dermal migration.

**Figure 5:**
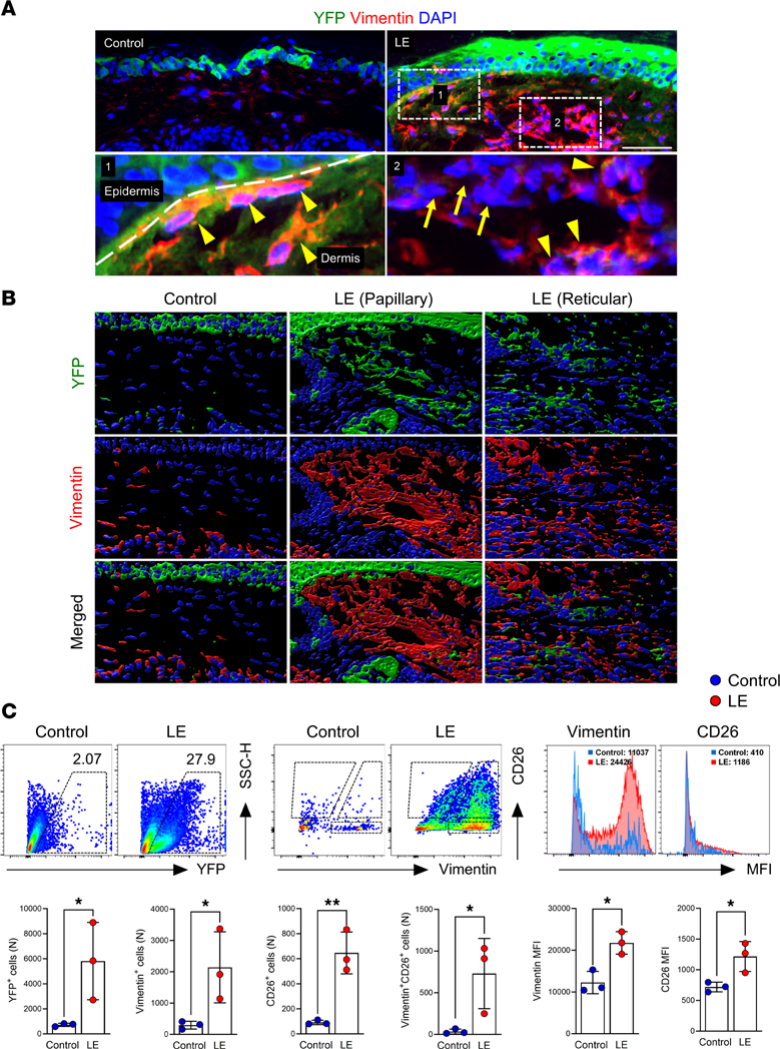
Lineage tracing confirms keratinocyte-derived fibroblasts in lymphedematous mouse tail skin. (**A**) Immunofluorescence images (scale bar: 100 μm) show YFP (green) and vimentin (red) staining in control and lymphedematous (LE) tail skin. Dashed lines delineate the epidermis and dermis. Magnified areas highlight YFP^+^ vimentin^+^ fibroblasts (arrowheads) and YFP^−^ vimentin^+^ fibroblasts (arrows) in LE skin. (**B**) 3D images of YFP and vimentin in control and LE skin underscore the increased presence and dermal distribution of YFP^+^ fibroblasts in lymphedema. (**C**) Representative flow cytometry plots and quantification graphs demonstrate a significant increase in YFP^+^ cells, vimentin^+^ CD26^+^ fibroblasts, and the mean fluorescence intensity (MFI) of vimentin and CD26 in the LE dermis compared with control. *P < 0.05, **P < 0.01 by unpaired Student’s *t* test. Error bars represent mean ± SD.

Tail skin and lymphatic excision in KRT14-YFP transgenic mice (tamoxifen-inducible expression of yellow fluorescent protein [YFP] under the regulation of the KRT14) resulted in localization of YFP^+^ cells only in the epidermis of control mice. In contrast, YFP^+^ cells were noted throughout the epidermis and dermis 6 weeks after surgery (Figure 5A). Dermal YFP^+^ cells also coexpressed vimentin (Figure 5B). Flow cytometry of isolated dermis corroborated these findings, showing increased YFP^+^ cells expressing fibroblast markers (vimentin, CD26, CD90, CD140α) in lymphedematous samples (Figure 5C and Supplemental Figure 2).

### TGF-β signaling drives keratinocyte EMT in lymphedema.

**Figure 6:**
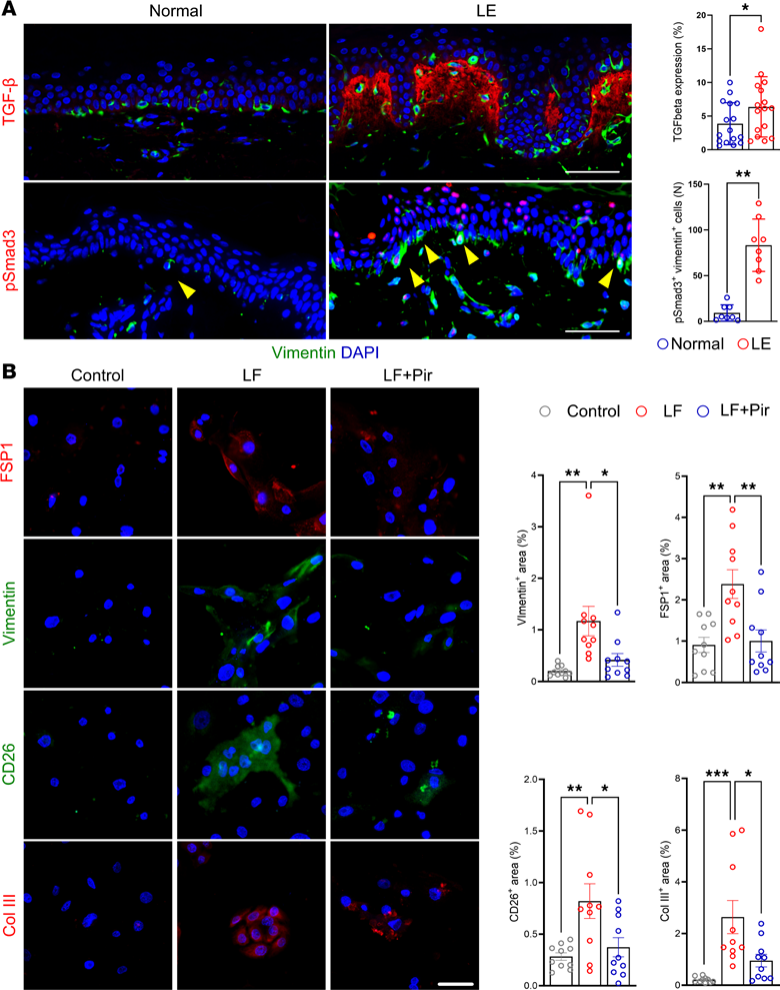
TGF-β signaling drives keratinocyte EMT in lymphedema, both in vivo and in vitro. (**A**) Immunofluorescence images (scale bar: 100 μm) show costaining for vimentin (green), TGF-β, and pSmad3 (red) in normal and lymphedematous (LE) skin. Dashed boxes highlight rete ridges. Quantification graphs show a significant increase in TGF-β and pSmad3 expression within vimentin^+^ cells in LE samples (*n* = 8–16). *P < 0.05, **P < 0.01 by paired Student’s *t* test. (**B**) Immunofluorescence images (scale bar: 20 μm) show staining for FSP1, vimentin, CD26, and collagen III in cultured human keratinocytes treated with phosphate buffered saline, LF, or LF with pirfenidone (Pir). Quantification graphs demonstrate LF-induced upregulation of EMT markers, which is significantly reduced by pirfenidone (*n* = 10). *P < 0.05, **P < 0.01, and ***P < 0.001 by 1-way ANOVA. Error bars represent mean ± SD.

TGF-β is a strong promoter of EMT and cellular invasiveness in other diseases. The expression of this growth factor is significantly increased in secondary lymphedema (28). Lymphedematous skin, particularly the rete ridges where vimentin^+^ keratinocytes cluster, showed significantly increased TGF-β expression (Figure 6A). Vimentin^+^ keratinocytes expressed pSmad3, indicating activation by TGF-β signaling. Lymph fluid (LF) from lymphedematous tissues contained TGF-β1, which induced the expression of FSP1, vimentin, CD26, and collagen III in cultured primary human keratinocytes (Supplemental Figure 3). Pirfenidone, a small-molecule inhibitor of TGF-β, significantly reversed these effects (Figure 6B).

**Figure 7:**
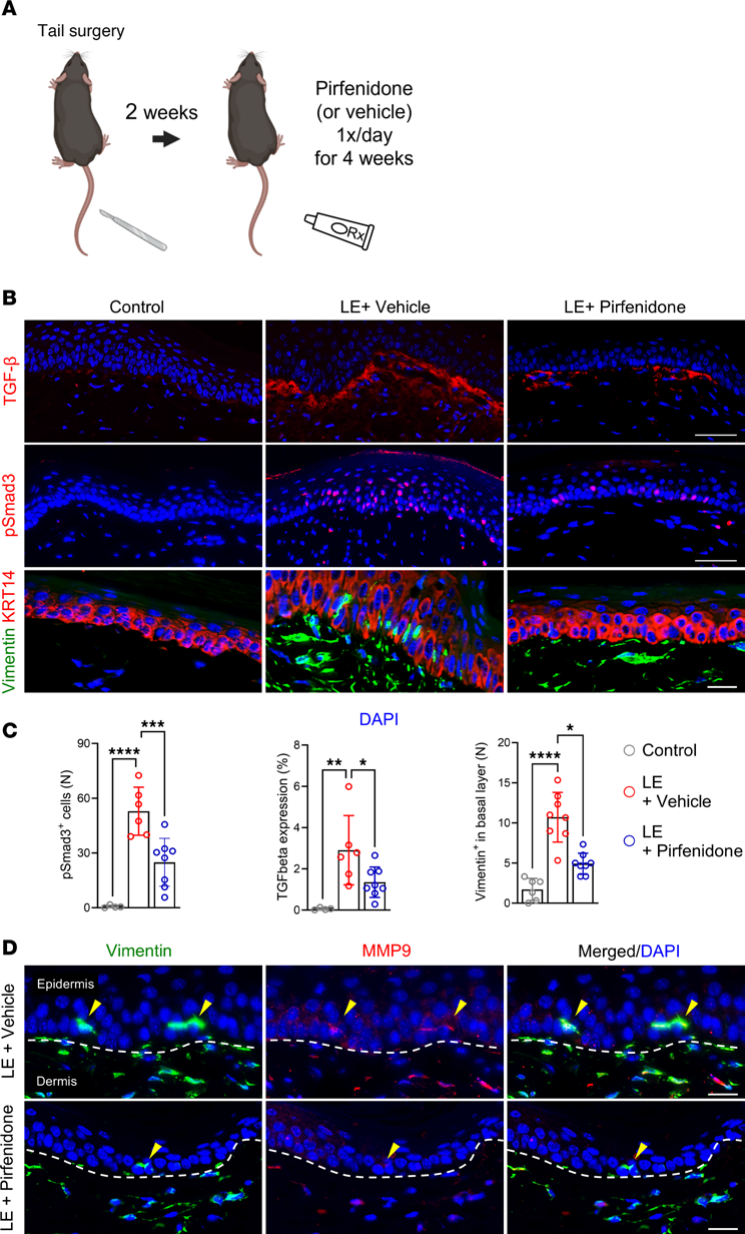
Topical pirfenidone reduces TGF-β signaling and keratinocyte EMT in lymphedematous mouse skin. (**A**) Schematic illustrates the timeline of tail skin/lymphatic excision, followed by 4 weeks of topical treatment with either vehicle (Aquaphor) or pirfenidone. (**B**) Immunofluorescence images (scale bar: 100 μm) show staining for TGF-β, pSmad3, vimentin, and KRT14 in control, lymphedematous (LE) + vehicle, and LE + pirfenidone groups. (**C**) Graphs quantify pSmad3^+^ cells, TGF-β–positive area, and vimentin^+^ cells (*n* = 4–8 mice). Significant reductions are seen in the pirfenidone-treated LE group. *P < 0.05, **P < 0.01, ***P< 0.001, ****P < 0.0001 by 1-way ANOVA. Error bars represent mean ± SD. (**D**) Immunofluorescence (scale bar: 100 μm) highlights vimentin (green) and MMP9 (red) in LE skin from vehicle and pirfenidone groups. Dashed lines delineate the epidermis and dermis. Arrowheads indicate vimentin^+^ cells with decreased MMP9 expression in the pirfenidone group.

We have previously shown that topical pirfenidone decreases skin fibrosis and improves lymphedema in the mouse tail model (11). Therefore, we next tested the efficacy of topical pirfenidone to decrease skin EMT in this model. As with our previous study, all animals underwent capillary and collecting lymphatic vessel excision and were allowed to recover for 2 weeks. Control mice were then treated with Aquaphor ointment and experimental animals were treated with Aquaphor containing 1% pirfenidone once a day for 4 weeks (Figure 7A) (11). Examination of skin in the pirfenidone-treated mice showed markedly decreased expression of TGF-β, a decreased number of pSmad3^+^ and vimentin^+^ keratinocytes, and decreased expression of MMP in vimentin^+^ keratinocytes (Figure 7, B–D). The expression of KRT14, a marker of keratinocyte differentiation, was highly abnormal in control mice as it was localized to cells in all the layers of the epidermis. In contrast, mice treated with topical pirfenidone had a much more normal-appearing expression pattern of KRT14 that was limited to the basal layer.

### Th2 cytokines regulate TGF-β and keratinocyte EMT.

**Figure 8:**
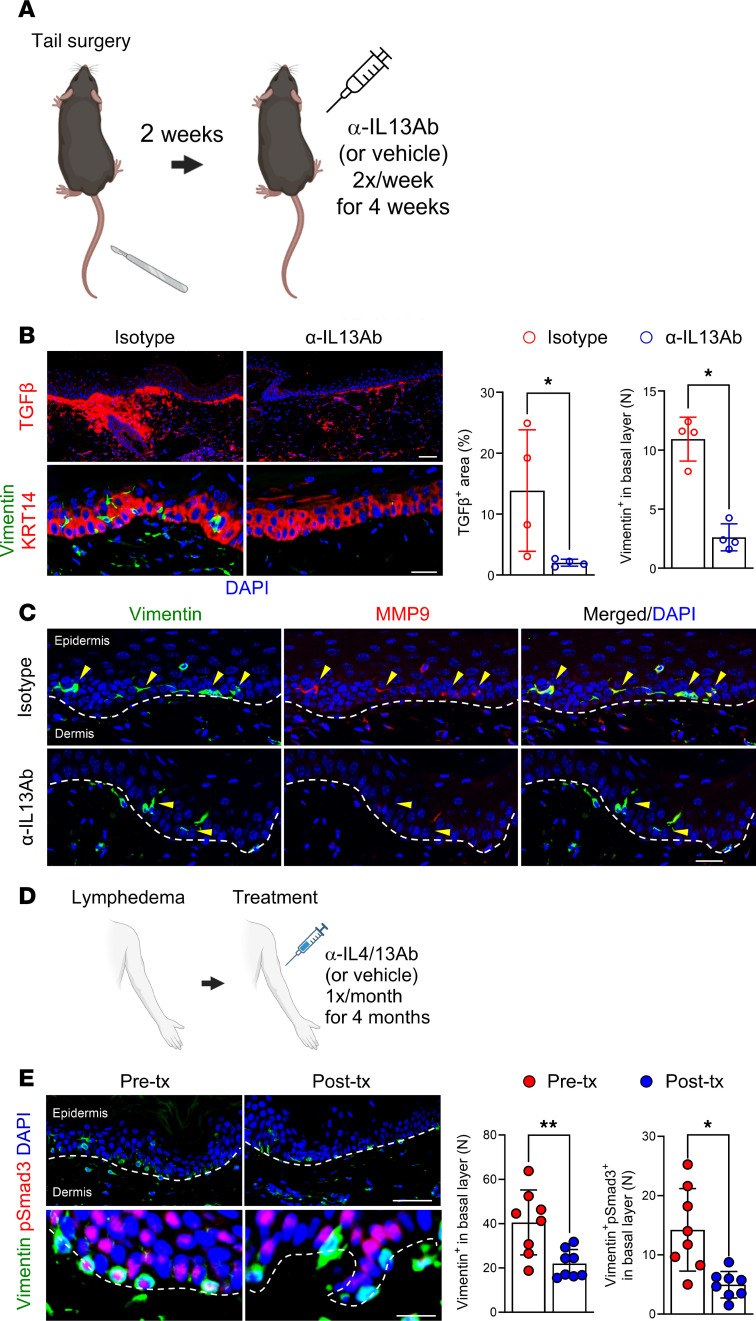
Th2 cytokine inhibition decreases TGF-β signaling and keratinocyte EMT in lymphedema. (**A**) Schematic illustration of the timeline of tail skin/lymphatic excision, followed by 4 weeks of treatment with either isotype control antibody or IL-13–neutralizing antibody (α-IL13). (**B**) Representative immunofluorescence images (scale bar: 100 μm) show TGF-β, vimentin, and KRT14 staining in control, lymphedematous (LE) + isotype, and LE + α-IL13 groups. Quantification graphs show significant reductions in TGF-β and vimentin^+^ cells in the α-IL13–treated LE skin (*N* = 4). *P < 0.05 by Mann-Whitney test. (**C**) Immunofluorescence (scale bar: 100 μm) highlights vimentin (green) and MMP9 (red) in LE + isotype and LE + α-IL13 groups. Dashed lines delineate the epidermis and dermis. Arrowheads indicate reduced MMP9 in vimentin^+^ cells following α-IL13 treatment. (**D**) Schematic illustrates the treatment regimen for patients with unilateral BCRL receiving QBX258 (anti–IL-4/IL-13) immunotherapy. (**E**) Immunofluorescence images (scale bar: 100 μm) show vimentin and pSmad3 staining in skin biopsies before and after QBX258 treatment. Dashed lines delineate the epidermis and dermis. Quantification graphs demonstrate a significant decrease in vimentin^+^ and pSmad3^+^ cells following therapy (*n* = 8). *P < 0.05, **P < 0.01 by paired Student’s *t* test. Error bars represent mean ± SD.

Inhibition of Th2 cytokines decreases TGF-β production in secondary lymphedema and other fibrotic diseases (17, 29). Therefore, to determine if Th2 cytokines regulate TGF-β–induced keratinocyte EMT, adult female mice underwent tail skin/lymphatic excision and were subsequently treated twice a week with isotype control antibody or IL-13–neutralizing monoclonal antibodies (5 μg/g) (Figure 8A) (17). IL-13 blockade significantly decreased TGF-β expression, reduced the number of vimentin^+^ keratinocytes, and decreased MMP9 expression in vimentin^+^ keratinocytes (Figure 8, B and C). Similar to topical pirfenidone, IL-13 neutralization normalized the expression of KRT14 in the epidermis.

We had previously performed a pilot study with 8 BCRL patients who were treated with anti-Th2 immunotherapy (QBX258; a combination treatment with monoclonal antibodies that block both IL-4 and IL-13) once a month for 4 months (Figure 8D) (12). This study showed that hyperkeratosis was decreased after immunotherapy; however, we had not assessed EMT. Therefore, in the current study, we reanalyzed skin biopsy samples obtained from our patient cohort before and 1 month after completion of a 4-month course of IL-4/IL-13–neutralizing antibodies. Consistent with our mouse model, this analysis showed a dramatic decrease in the number of vimentin^+^ and pSmad3^+^ keratinocytes in the epidermis (Figure 8E).

## Discussion

In this study, we showed that keratinocytes undergo EMT in secondary lymphedema and contribute to the process of skin fibrosis. Using clinical biopsy samples and a mouse model, we observed distinct hallmarks of EMT in lymphedematous skin: epidermal thickening, elongated rete ridges, and a significantly increased number of vimentin^+^ cells in the basal epidermis. Importantly, these vimentin^+^ cells exhibited loss of epithelial markers and upregulation of mesenchymal markers, along with evidence of migration into the dermis. Lineage tracing in transgenic mice confirmed the keratinocyte origin of these dermal fibroblasts, and in vivo and in vitro studies showed that TGF-β and Th2 cytokines play important roles in this process. Our findings are supported by previous reports demonstrating that EMT is an important regulator of fibrosis in a variety of clinical scenarios, including inflammatory skin disorders such as atopic dermatitis and psoriasis (30–34).

Our findings suggest that TGF-β signaling in the papillary dermis plays a pivotal role in driving keratinocyte EMT in lymphedema. This is consistent with a previous study showing that skin dermal papillae create a TGF-β–rich microenvironment that supports interaction between fibroblasts and keratinocytes/hair follicles (35). We observed increased expression of TGF-β in the dermal papillae, particularly near the rete ridges, along with the accumulation of vimentin^+^ epithelial cells expressing pSmad3. Furthermore, LF from lymphedematous tissue induced EMT in cultured keratinocytes, an effect that was blocked by TGF-β inhibition with pirfenidone. This aligns with the established role of TGF-β as a potent inducer of EMT in various fibrotic diseases (22, 36, 37).

We also elucidated a key interplay between Th2 cytokines and TGF-β signaling in the context of lymphedematous fibrosis. These findings are consistent with previous reports in other organ systems demonstrating bidirectional interaction between TGF-β and Th2 cytokines in fibrotic disorders (38–42). In our mouse model, inhibiting IL-13 reduced TGF-β expression and keratinocyte EMT. Analysis of clinical biopsy samples following anti-Th2 immunotherapy corroborated these findings, revealing a significant decrease in vimentin^+^ and pSmad3^+^ keratinocytes. Together, these findings support the hypothesis that Th2 cytokines positively regulate TGF-β expression in lymphedema and that the combined expression of these cytokines modulates keratinocyte EMT.

Our results highlight the multifaceted role of keratinocytes in lymphedema pathology. Beyond the recognized function of keratinocytes in modulating Th2 inflammatory responses by producing Th2-inducing cytokines such as TSLP and IL-33 (43), this study underscores the importance of these cells to the fibrotic process via EMT. This expands our understanding of secondary lymphedema and suggests that targeting keratinocytes could be a promising therapeutic avenue.

Our study has some limitations. LF was collected from stage II lymphedema patients since stage I lymphedema patients do not have much excessive tissue fluid in general. Similarly, patients with stage III disease usually do not have significant accumulation of LF but rather have extensive fibroadipose tissue deposition. It is also impossible to obtain enough extracellular fluid from normal tissues for in vitro studies, thus limiting our controls for experiments in which we used LF. However, we tried to mitigate this issue by obtaining LF from 6 different patients to bolster our findings. In addition, pirfenidone prevented fibrosis and EMT when administered in the early postoperative period, suggesting that TGF-b plays an important role in this process. Future studies are needed to determine if TGF-b blockade can reverse established EMT in late-stage lymphedema.

**Figure 9:**
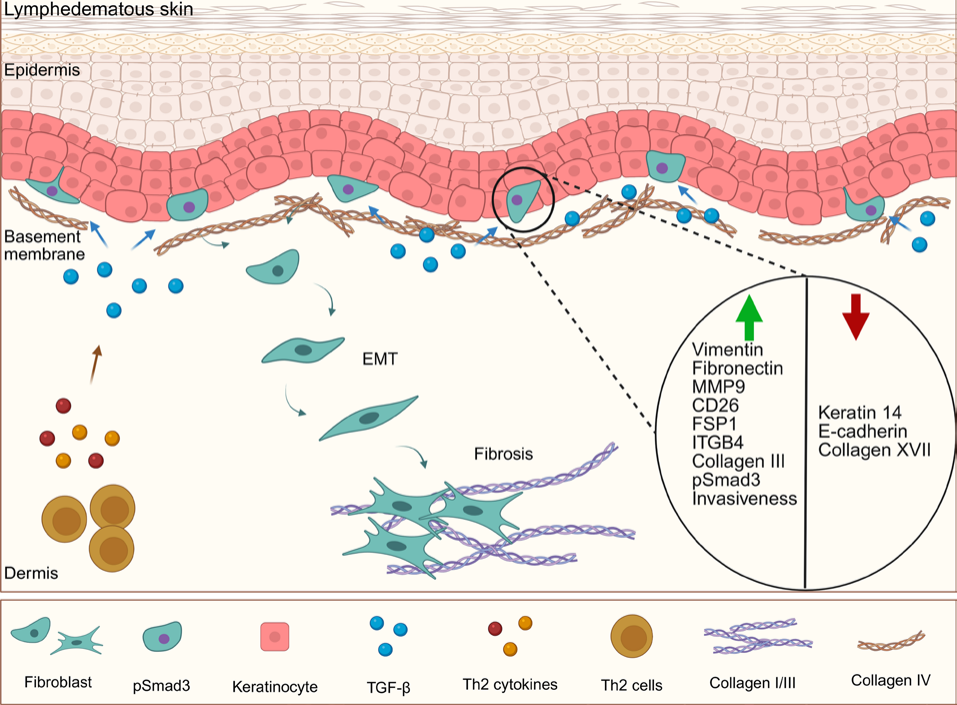
Proposed model of TGF-β–driven keratinocyte EMT and dermal fibrosis in lymphedema. Lymphedema creates a profibrotic environment with elevated dermal TGF-β, potentially regulated by Th2 cytokines. TGF-β induces EMT in keratinocytes in the basal layer of the epidermis. These cells exhibit increased mesenchymal markers and decreased epithelial markers, and they migrate into the dermis. Transformed keratinocytes differentiate into fibroblasts, contributing to dermal fibrosis through the production of ECM.

In conclusion, this study identifies previously unknown keratinocyte EMT as a novel mechanism contributing to skin fibrosis in secondary lymphedema (Figure 9). Our findings implicate a signaling axis involving TGF-β within the dermal papillae, potentially regulated by Th2 cytokines, that promotes keratinocyte EMT and subsequent fibroblast transformation.

## Methods

### Sex as a biological variable.

This study involves human biopsy specimens and interstitial lymphedema fluid from women with upper extremity breast cancer–related lymphedema (BCRL). Treatment studies utilized adult (8–12 week-old) female C57BL/6J mice. The exclusion of male human and mouse specimens was due to the higher prevalence of BCRL in females, necessitating further research to determine the relevance of these findings to the male sex.

### Clinical lymphedema biopsy specimens.

Women with unilateral upper extremity BCRL were identified at our lymphedema clinic and screened for eligibility for harvesting of biopsy specimens. Inclusion criteria included age between 21 and 75 years, unilateral axillary surgery, and stage I–II lymphedema (volume differential of > 10% with the normal limb or L-Dex measurements above 7.5 units). Exclusion criteria included pregnant or lactating women, recent (within 3 months) history of lymphedematous limb infection, chemotherapy, treatment with steroids or other immunosuppressive agents, and active cancer or breast cancer metastasis. We harvested excess interstitial LF from the lymphedematous arm and 5-mm full-thickness skin biopsies from the volar surface of the normal and lymphedematous limb at a point located 5–10 cm below the elbow crease. Surgery was performed under sterile conditions with local anesthesia. Patients were treated with a dose of antibiotics (1,000 mg cephalexin or 600 mg clindamycin if penicillin-allergic) 30–60 minutes before the procedure. Study design for anti-Th2 immunotherapy is described in a previous publication (12).

### Animals.

Adult (8–12 week-old) female C57BL/6J mice were used for all treatment studies. We used female mice because secondary lymphedema affects females more commonly than males. KRT14Cre mice were bred with YFP floxed C57BL/6J mice (a gift from Elaine Fuchs, The Rockefeller University, New York, NY, USA). Per the IACUC-approved protocol, all mice were maintained in light- and temperature-controlled pathogen-free environments and fed ad libitum.

### Surgical models of lymphedema.

Anesthesia was induced using isoflurane (Henry Schein Animal Health, Dublin, OH, USA), and mice were kept on a heating blanket to maintain body temperature. Depth of anesthesia was monitored by reaction to pain and observation of respiratory rate. Animals were excluded from the experiment if wound infection or ulceration in the tail was noted at any time point following surgery. Postoperative pain control was maintained with 3 doses of intraperitoneal buprenorphine injection every 4–12 hours. Animals were euthanized by carbon dioxide asphyxiation as recommended by the American Veterinary Medical Association.

We used 2 well-described mouse models of lymphedema (44). In the tail surgery model, both the superficial and deep lymphatic vasculature were ligated through a 2-mm circumferential excision of the skin, 2 cm distal to the base of the tail. Collecting lymphatics were identified using Evans blue injection and ligated along the entire length of the skin excision. Control animals underwent skin incision without lymphatic ligation (17, 45).

### Histology and immunofluorescence.

Histological and immunofluorescence analyses were performed using our previously published techniques (16, 46, 47). Clinical and experimental biopsy specimens were fixed in 4% paraformaldehyde (Sigma-Aldrich, St. Louis, MO, USA) overnight. Tails were decalcified using 5% ethylenediaminetetraacetic acid (Santa Cruz Biotechnology, Santa Cruz, CA, USA), embedded in paraffin, and sectioned at 5 μm. Hematoxylin and eosin (H&E) staining was performed using standard techniques. For immunofluorescent staining, the rehydrated sections underwent heat-mediated antigen unmasking with sodium citrate (Sigma-Aldrich) and quenching of endogenous peroxidase activity. The sections were then incubated at 4 °C with the appropriate primary antibodies overnight. The list of antibodies utilized is in Table 1.

H&E and IHC slides were evaluated with brightfield or fluorescent microscopy and scanned using a Mirax slide scanner (Carl Zeiss, Oberkochen, Germany). Staining was visualized using Slide Viewer (3DHISTECH Ltd., Budapest, Hungary). The epidermal area was quantified in H&E-stained tail cross sections by measuring the ratio of dark-stained epidermis within the total tissue area using MetaMorph Offline software (Molecular Devices, Sunnyvale, CA, USA) with a minimum of 4 high-powered fields per tissue by 2 blinded reviewers. Cell counts were quantified in IHC-stained tail cross sections by counting the cells with positive staining. The protein-expressing area was quantified as a ratio of the area of positively stained dermis within a fixed threshold to the total tissue area using MetaMorph Offline software (Molecular Devices) with a minimum of 4 high-powered fields per animal by 2 blinded reviewers.

### RNA-seq.

RNA-seq was performed in collaboration with the Integrated Genomics Operation (IGO) Core Facility at MSK. Four pairs of frozen clinical lymphedema biopsy specimens were submitted to the IGO. The ribodepletion method was used for RNA-seq. mRNA expression was standardized and analyzed by the IGO. Standardized expression for each molecule was assessed and data are presented as Z-scores.

### Western blot analysis.

Clinical and mouse skin biopsies were frozen in liquid nitrogen and homogenized. The samples were then lysed with a radioimmunoprecipitation assay lysis buffer containing the single-use Halt Protease and Phosphatase Inhibitor Cocktail (Thermo Fisher Scientific, Waltham, MA, USA). The lysates were centrifuged at 13,000*g* for 10 minutes at 4 °C, and the protein concentration was measured by a BCA protein assay kit (Thermo Fisher Scientific) according to the manufacturer’s directions. Total protein (1–20 μg) was separated by NuPAGETM 4–12% Bis-Tris gel (Thermo Fisher Scientific) and transferred onto polyvinylidene difluoride membranes (Bio-Rad Laboratories, Inc., Hercules, CA, USA). The membranes were blocked with 5% skim milk in TBS containing 0.1% Tween 20 (TBST) at room temperature for 1 hour and incubated with antibodies against fibronectin (ab2413; Abcam, Cambridge, UK), MMP9 (ab38898; Abcam), CD25 (AF954; R&D Systems, Inc., Minneapolis, MN, USA), vimentin (ab137321; Abcam), FSP1 (ABF32; Sigma-Aldrich), collagen III (22734-1-AP; Proteintech Group, Inc., Rosemont, IL, USA), and glyceraldehyde-3-phosphate dehydrogenase (GAPDH; MAB374; Sigma-Aldrich) in 0.5% skim milk in TBST at 4 °C overnight. After washing 3 times with TBST, the membranes were incubated with horseradish peroxidase–conjugated secondary antibody in TBST at room temperature for 1 hour. The membranes were then washed with TBST, and immune-reactive bands were detected with ECL Western Blotting Substrate (Thermo Fisher Scientific). Protein expression was quantified with ImageJ software (National Institutes of Health, Bethesda, MD, USA) and normalized to housekeeping genes, GAPDH, or β-actin.

### Flow cytometry.

Flow cytometry was performed to quantify inflammation in the mouse tails after tail surgery (48). Briefly, single-cell suspensions were obtained from a 3-cm portion of the tail distal to the surgical site using a combination of mechanical dissociation and enzymatic digestion with a solution of DNase I, Dispase II, collagenase D, and collagenase IV (all Roche Diagnostics, Indianapolis, IN, USA) mixed in 2% fetal calf serum (Sigma-Aldrich). Cells were stained with combinations of the following antibodies: goat anti-YFP (AB1166-100; OriGene Technologies, Inc., Rockville, MD, USA), donkey anti-goat FITC (A16006; Invitrogen, Waltham, MA, USA), rat anti-vimentin Alexa Fluor 647 (699307; BioLegend, San Diego, CA, USA), rat anti-CD140a BV605 (135916; BioLegend), rat anti-CD90.2 APC Cy7 (140331; BioLegend), and rat anti-CD26 PE (137803; BioLegend). Intracellular staining was performed with BD Cytofix/Cytoperm (AB2869008; BD Biosciences, Franklin Lakes, NJ, USA). DAPI viability stain was also used on all samples to exclude dead cells. Single-stain compensation samples were created using UltraComp eBeads (01-2222-42; Applied Biosystems, Waltham, MA, USA). Flow cytometry was performed using a BD Fortessa flow cytometry analyzer (BD Biosciences) with BD FACS Diva, and data were analyzed with FlowJo software (BD Biosciences).

### In vitro keratinocyte culture.

Human keratinocytes (PCS-200-011; American Type Culture Collection [ATCC], Manassas, VA, USA) were cultured in dermal cell basal medium (PCS-200-030; ATCC) with a keratinocyte growth kit (PCS-200-040; ATCC). Keratinocytes were cultured with or without 10% lymphedema fluid in keratinocyte medium for 72 hours.

### Pirfenidone treatment.

A topical formulation of pirfenidone (PFD; 1% PFD dissolved in Aquaphor; Beiersdorf, Hamburg, Germany) was developed in collaboration with the Research Pharmacy Core Facility at MSK. This dose of PFD was based on previous studies showing effective treatment regimens for various fibrosis models (49–51). The control group was treated with Aquaphor alone. PFD or Aquaphor treatment was initiated 2 weeks after tail surgery. The treatment was applied once daily for 4 weeks to the tail region distal to the zone of lymphatic/skin excision.

### Antibodies and inhibitors.

We used a well-described monoclonal rat antibody against mouse IL-13 (5 μg/g; clone 38213; R&D Systems, Inc.) administered intraperitoneally (29). Controls were treated with similar doses of isotype control antibodies (Bio X Cell, Lebanon, NJ, USA). To determine the role of IL-13 in the EMT of keratinocytes, C57BL/6J mice were treated with IL-13 monoclonal antibody or isotype control antibodies 24 hours before and then every 4 days for 6 weeks after surgery.

### Anti-Th2 immunotherapy study design and approval.

This pilot study was approved by the Institutional Review Board at MSK and previously described (12). Patients diagnosed with unilateral stage I or II BCRL received QBX258, which is a combination of 2 humanized monoclonal antibodies that inhibit the bioactivity of IL-4 and IL-13.

### Statistics.

Statistical analysis was performed using GraphPad Prism software (Dotmatics, Boston, MA, USA). Samples were assessed for normal distribution using the Shapiro-Wilk test. Normally distributed clinical samples were analyzed using a paired Student’s *t* test. Comparison of multiple groups or time points was performed using an unpaired Student’s *t* test, a Mann-Whitney test, 1-way ANOVA) or 2-way ANOVA with multiple comparisons using Tukey’s multiple comparison test. Data are presented as mean ± SD unless otherwise noted, with *P* < 0.05 considered significant. For all figures, each dot represents 1 animal or patient unless noted otherwise.

### Study approval.

All procedures involving human participants were approved by the Institutional Review Board at Memorial Sloan Kettering Cancer Center (MSK). All studies involving vertebrate animals were approved by the Institutional Animal Care and Use Committee (IACUC) at MSK under protocol 06-08-018. The MSK IACUC adheres to the National Institutes of Health Public Health Service Policy on Humane Care and Use of Laboratory Animals and operates in accordance with the Animal Welfare Act and the Health Research Extension Act of 1985.

### Data availability.

All the RNA-seq raw data has been deposited in GEO under accession number GSE302118. Raw data for this manuscript have been provided as a Supporting data values file.

## Author contributions

HJP, BJM, and RPK conceptualized the study. HJP and RPK developed the methodology. HJP, RPK, JS, AS, and SP conducted the experiments. MC, JHD, and BJM collected the lymphedema human biopsies. MGK formulated and prepared the topical pirfenidone. ER helped and oversaw the statistical methods. BJM and RPK acquired the funding. HJP and RPK wrote the original draft. HJP, RPK, and BJM participated in the editing and review of the manuscript.

## Supplementary Material

Supplemental data

Unedited blot and gel images

Supporting data values

## Figures and Tables

**Table 1:**
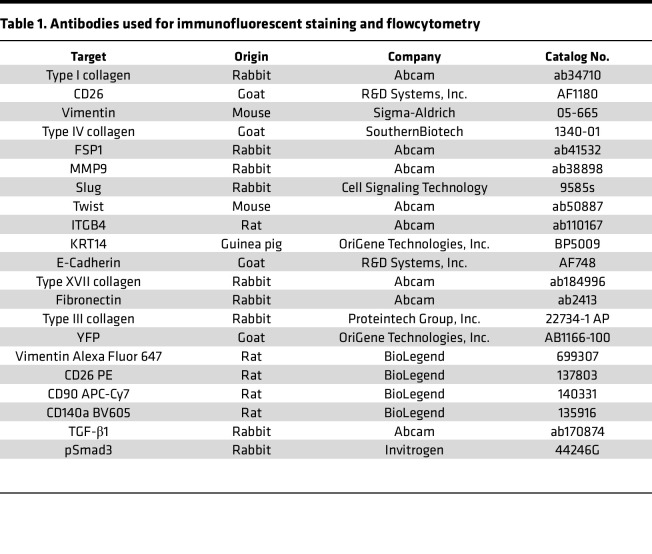
Antibodies used for immunofluorescent staining and flowcytometry

## Version 1 07/29/2025

In-Press Preview

## Version 2 09/09/2025

Electronic publication
