# Supplementary material for: TGF-β–mediated epithelial-mesenchymal transition of keratinocytes promotes fibrosis in secondary lymphedema
Source: JCI Insight. 2025 Jul 29;10(17):e192890. doi: 10.1172/jci.insight.192890 (PMC12487678; doi:10.1172/jci.insight.192890)
Supplement: Supplemental data [file jciinsight-10-192890-s136.pdf]

TGF-β–mediated epithelial–mesenchymal transition of keratinocytes promotes fibrosis in secondary lymphedema.

Supplementary data:

	Value
Patients (n)	4
Age (years)	60.2 ± 8.3
Baseline BMI (Kg/mg^2)	24.1 ± 3.6
ISL stage	
Stage I	1
Stage II	3
Volume differential (%)	13.4 ± 0.05
Volume differential (cc)	245.7 ± 95.7
L-Dex Score	19.4 ± 7.7
Dutation (months)	90.7 ± 92.7
Radiation (n)	4/4 (100%)
Cellulitis episode/year	6.5 ± 7.2

Supplementary table 1. Demographics of RNAseq analysis performed patients (n=4). Data are presented as mean ± standard deviation unless noted. BMI, body mass index; ISL, International Society of Lymphology.

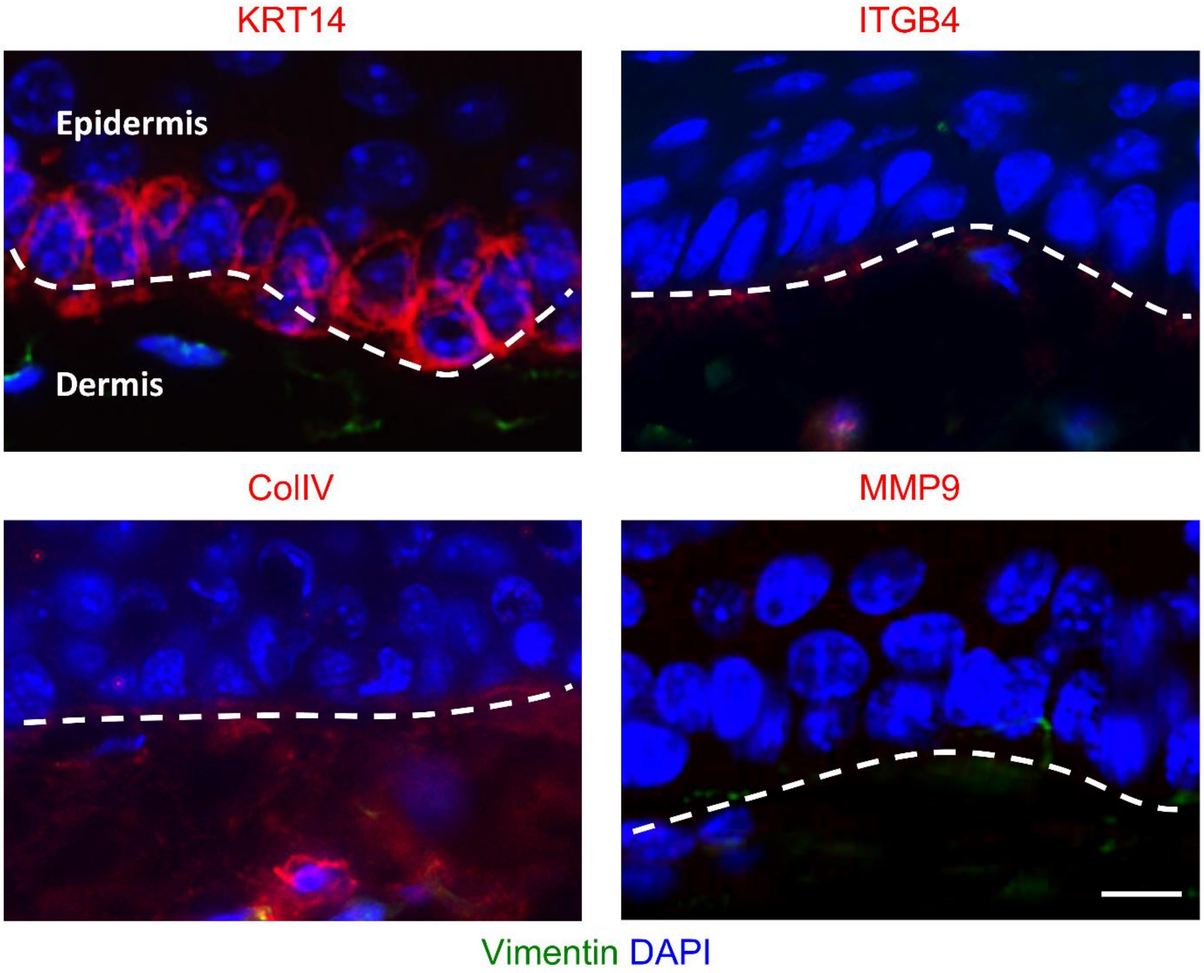

Supplementary Figure 1. Absence of EMT markers in control mouse tail skin.

Immunofluorescent images (scale bar, 100 µm) of control mouse tail skin harvested 6 weeks post-surgery. Staining shows vimentin (green) along with EMT markers and the basement membrane marker collagen IV (red). Dashed lines delineate the epidermis and dermis. Note the

lack of vimentin^{+} cells within the epidermis and the minimal co-expression of vimentin with EMT markers.

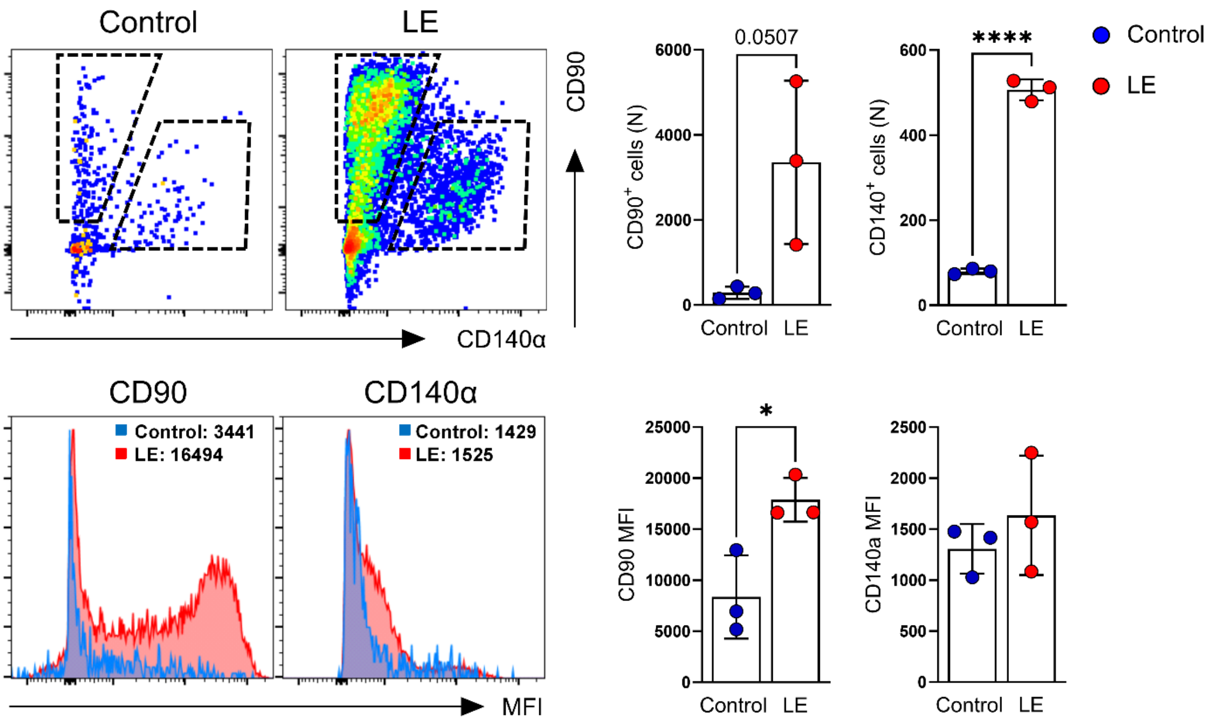

Supplementary Figure 2. YFP^{+} cells in lymphedematous dermis express fibroblast markers. Flow cytometry analysis of isolated dermal cells from control and lymphedematous mouse tail skin. Top: Quantification of CD90^{+} and CD140α^{+} fibroblasts in YFP^{+} cell populations. Bottom: Mean fluorescence intensity (MFI) of vimentin and CD26 in YFP^{+} dermal cells. *$p < 0.05$ and ****$p < 0.0001$ by unpaired Student's *t* test.

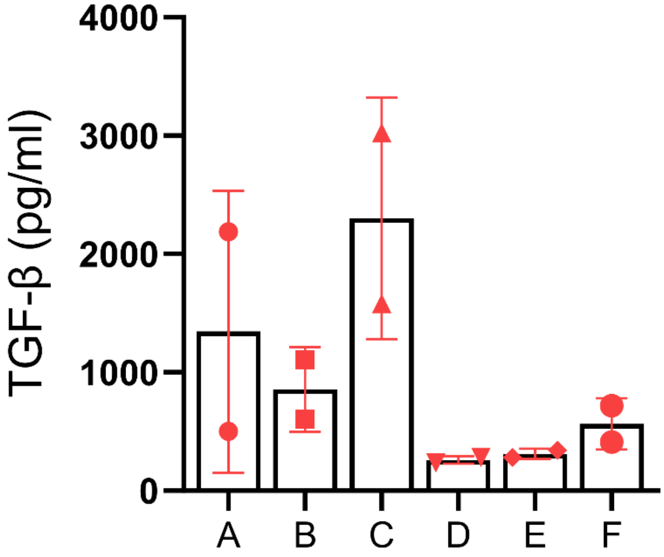

Supplementary Figure 3. Lymph fluid from BCRL patients contains TGF-β. ELISA quantification of TGF-β in lymph fluid from patients with unilateral BCRL.
