# Supplementary material for: TGF-β–mediated epithelial-mesenchymal transition of keratinocytes promotes fibrosis in secondary lymphedema
Source: JCI Insight. 2025 Jul 29;10(17):e192890. doi: 10.1172/jci.insight.192890 (PMC12487678; doi:10.1172/jci.insight.192890)

GAPDH: MAB374(Sigma)

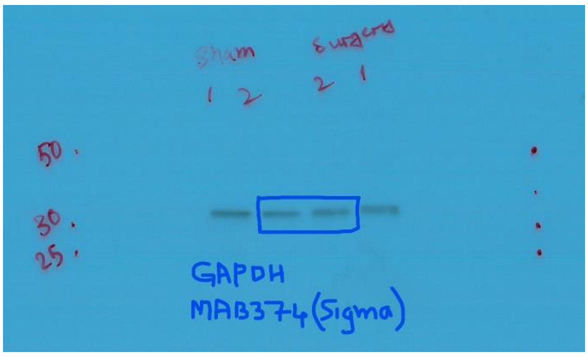

Fibronectin: ab2413(abcam)

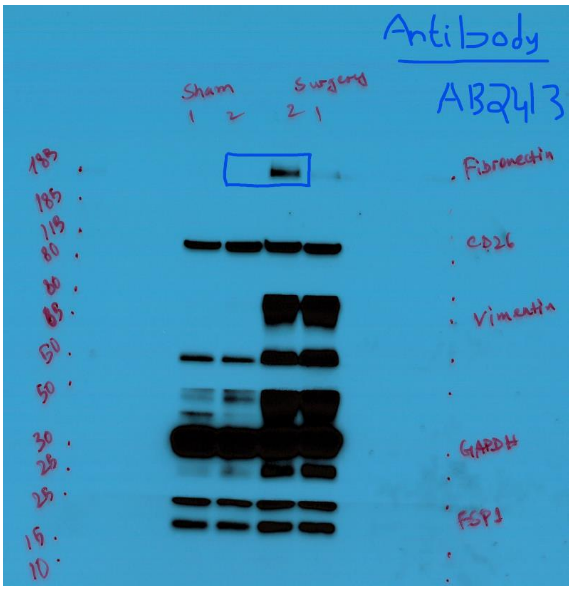

Collagen III: 22734-1-AP(proteintech)

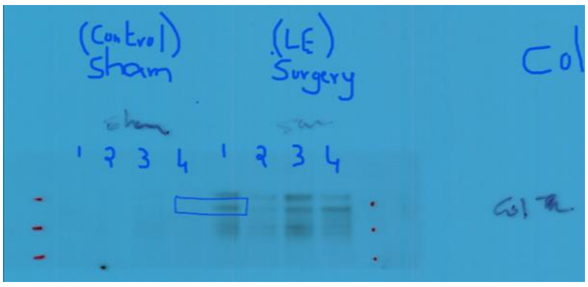

MMP9: ab38898(abcam)

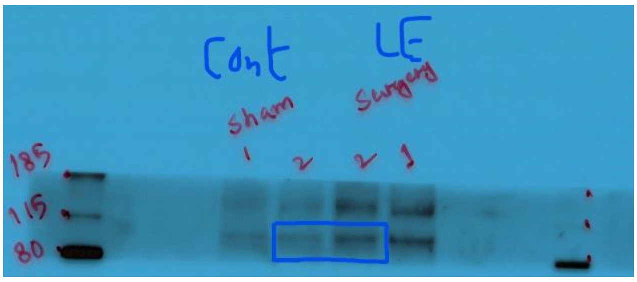

CD26: AF954(R&D)

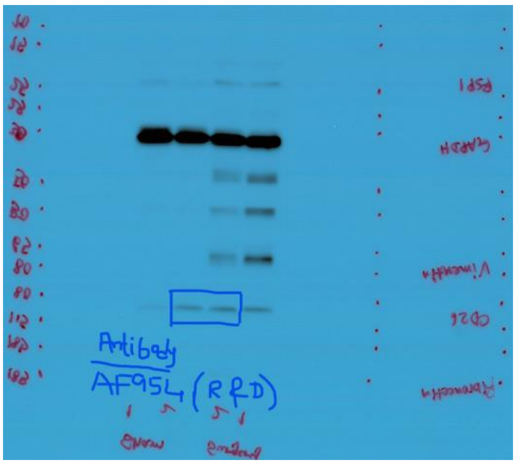

Vimentin: ab137321(abcam)

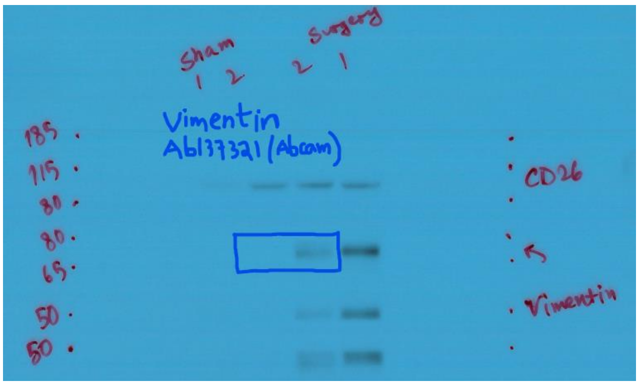

FSP1: ABF32(Sigma)

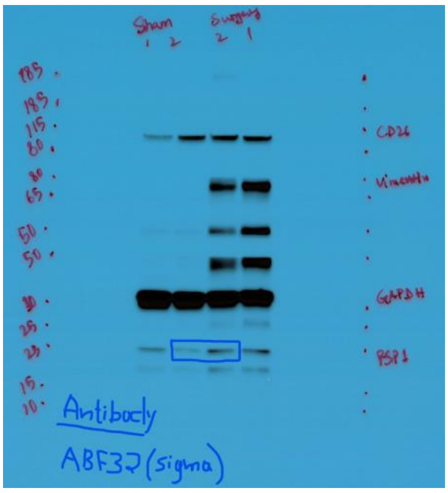

Supplement: Unedited blot and gel images [file jciinsight-10-192890-s137.pdf]
